# Volume Matters in Ultrasound-Guided Perineural Dextrose Injection for Carpal Tunnel Syndrome: A Randomized, Double-Blinded, Three-Arm Trial

**DOI:** 10.3389/fphar.2020.625830

**Published:** 2020-12-17

**Authors:** Meng-Ting Lin, Chun-Li Liao, Ming-Yen Hsiao, Hsueh-Wen Hsueh, Chi-Chao Chao, Chueh-Hung Wu

**Affiliations:** ^1^ Department of Physical Medicine and Rehabilitation, National Taiwan University Hospital Hsin-Chu Branch, Hsinchu, Taiwan; ^2^ Department of Physical Medicine and Rehabilitation, National Taiwan University Hospital, College of Medicine, National Taiwan University, Taipei, Taiwan; ^3^ Department of Neurology, National Taiwan University Hospital, Taipei, Taiwan; ^4^ Department of General Medicine, National Taiwan University Hospital Biomedical Park Branch, Hsinchu, Taiwan

**Keywords:** median nerve, injection, hydrodissection, dextrose, entrapment neuropathy

## Abstract

Ultrasound-guided perineural dextrose injection (PDI) has been reported effective for carpal tunnel syndrome (CTS). Higher volume of injectate may reduce adhesion of median nerve from other tissues, but volume-dependent effects of PDI in CTS remain unknown. We aimed to investigate whether PDI with different injectate volumes had different effects for CTS participants. In this randomized, double-blinded, three-arm trial, 63 wrists diagnosed with CTS were randomized into three groups that received ultrasound-guided PDI with either 1, 2 or 4 ml of 5% dextrose water. All participants finished this study. Primary outcome as visual analog scale (VAS) and secondary outcomes including Boston Carpal Tunnel Questionnaire (BCTQ), Disability of the Arm, Shoulder and Hand score (QuickDASH), electrophysiological studies and cross-sectional area (CSA) of the median nerve at carpal tunnel inlet were assessed before and after PDI at the 1st, 4th, 12th and 24th weeks. For within-group analysis, all three groups (21 participants, each) revealed significant improvement from baseline in VAS, BCTQ and QuickDASH at the 1st, 4th, 12th and 24th weeks. For between-group analysis, 4 ml-group yielded better VAS reduction at the 4th and 12th weeks as well as improvement of BCTQ and QuickDASH at the 1st, 4th, and 12th weeks, compared to other groups. No significant between-group differences were observed in electrophysiological studies or median nerve CSA at any follow-up time points. There were no severe complications in this trial, and transient minor adverse effects occurred equally in the three groups. In conclusion, ultrasound-guided PDI with 4 ml of 5% dextrose provided better efficacy than with 1 and 2 ml based on symptom relief and functional improvement for CTS at the 1st, 4th, and 12th week post-injection, with no reports of severe adverse effects. There was no significant difference between the three groups at the 24th-week post-injection follow-up.

**Clinical Trial Registration:** www.ClinicalTrials.gov, identifier NCT03598322.

## Introduction

Carpal tunnel syndrome (CTS), the most common compressive mononeuropathy caused by entrapment of the median nerve, leads to functional impairment of the hand (Atroshi et al., 1999). Possible etiologies include increased pressure in the intracarpal canal, which compromise the circulation of median nerve (Bland, 2005) and tissue adhesion around median nerve (LaBan et al., 1986; Smith et al., 2008). As for treatment, wrist resting splint and steroid injection have long been the mainstream of conservative treatments, while surgical intervention was reserved for severe or refractory cases (Huisstede et al., 2014). Recently, however, perineural dextrose injection (PDI) has been reported to be beneficial or even better than steroid injection (Wu et al., 2017b; Wu et al., 2018). To explain the therapeutic response of PDI with the hypoosmolar 5% dextrose for neuropathy-related pain, sensorineural mechanism was postulated that analgesic effect of dextrose on tender peripheral nerves as well as central nerve system via caudal epidural injection (Maniquis-Smigel et al., 2016). Potential down-regulation to molecular pathway on the ion channel capsaicin receptor of sensory neurons may attenuate nociceptive and neuropathic pain (Watabiki et al., 2011; Bertrand et al., 2015).

PDI pertains to nerve mobilization, a broader proposition for CTS treatment, which facilitates adhesion release (Page et al., 2012). Perineural injection to separate the median nerve from the flexor retinaculum and flexor tendons at the carpal tunnel inlet with normal saline was reported beneficial to CTS (Wu et al., 2019). Nerve mobilization or hydrodissection implies that adhesiolysis of the median nerve from surrounding tissues may play a role in treating CTS. In clinical practice, we also observed better detachment of the median nerve from surrounding tissues in the carpal tunnel with a larger injecting volume.

However, previous studies mostly focused on injectate content (steroid, 5% dextrose water, platelet-rich plasma, etc.), where the injecting volumes vary between studies (from 1 to 5 ml) (Armstrong et al., 2004; Peters-Veluthamaningal et al., 2010; Atroshi et al., 2013; Wu et al., 2017a; Wu et al., 2018). In other words, the effect of injecting volume itself was not investigated, and the optimal amount has yet to be determined. In this randomized, double-blinded, three-arm trial, we aimed to investigate whether PDI with different injectate volumes has different effects for CTS participants.

## Methods

### Study Design

This study was a prospective, parallel three-arm, double-blinded randomized control trial, approved by IRB of our Hospital. The study was conducted in accordance with the Declaration of Helsinki. The enrollment started in June 2018, and all follow-ups were completed by December 2019. All participants provided written informed consent. The participants and the outcome assessor were blinded for treatment allocation, while the physiatrist performing the injection was the only one aware of the treatment allocations.

### Sample Size

A preliminary power analysis using G-power 3.1.9.4 (University of California, Los Angeles) was calculated in an analysis of variance for comparison of three groups. For an effect size of 0.41 (1 − *β*) = 0.8 and *α* = 0.05, data for at least 60 wrists were required to achieve sufficient power.

### Inclusion and Exclusion Criteria

Inclusion criteria were 1) aged 20–80 years and 2) diagnosed with idiopathic CTS. The participants were required to fulfill the electrophysiological criteria and at least one of the symptoms and signs which are to be described. Abnormal electrophysiological analysis was defined as at least one of the following criteria: 1) a distal motor latency of the median nerve of more than 3.6 ms at a distance of approximately 7 cm from the abductor pollicis brevis muscle with median nerve stimulation at the wrist; 2) a sensory nerve conduction velocity (SNCV) from digit-to-wrist segment (14 cm) of less than 40 m/s or an SNCV from mid-palm-to-wrist segment (8 cm) of less than 37 m/s. Symptoms and signs included 1) pain or paresthesia in the median nerve innervated area (at least two digits with such symptoms between the thumb and the 4th digit) for more than 2 months; 2) positive Phalen test, Tinel sign, or flick sign (Chang et al., 2008; Atroshi et al., 2013). Exclusion criteria were previous wrist surgery, traumatic wrist injury within 2 years, previous wrist injection within 3 months, history of peripheral nerve injuries (brachial plexopathy, cervical radiculopathy or thoracic outlet syndrome), history of thyroid or autoimmune disease, and inability to cooperate with study protocol.

### Randomization

**FIGURE 1:**
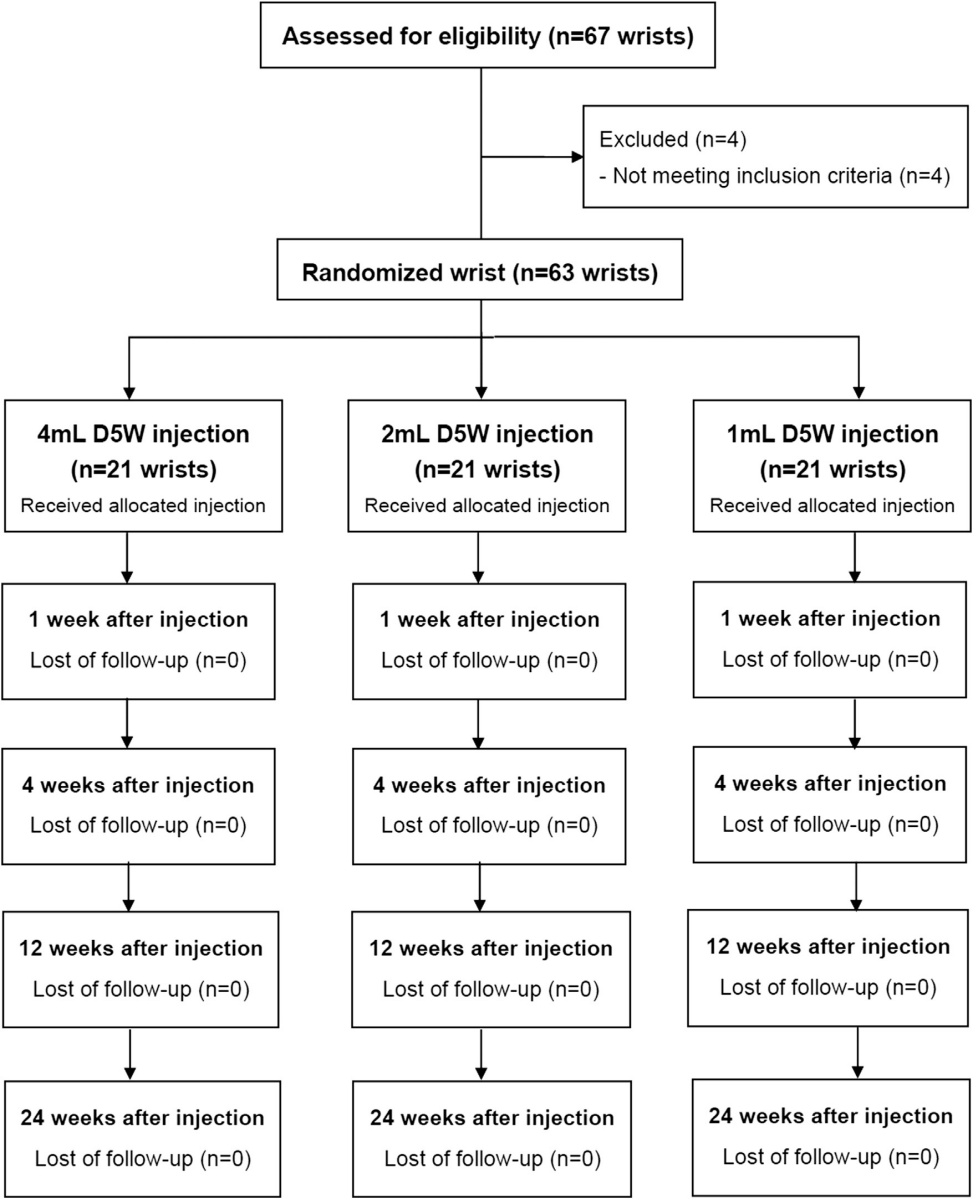
Study flow diagram. D5W, 5% dextrose water.

Among the 43 participants with 67 wrists that were assessed for eligibility into the trial, four participants were excluded, as they did not meet the inclusion criteria. Therefore, a total of 63 wrists were randomized into three groups, each with 21 wrists (Figure 1). We performed randomization in permuted blocks of six, and the independent research assistant, who was not involved in the eligibility selection process of participants, prepared and sealed the covered envelopes containing the intervention allocation. The participants were assigned to either 1-ml group, 2-ml group or 4-ml group and all of them received a corresponding session of ultrasound-guided PDI. After the injection, all participants were allowed to take simple analgesics (paracetamol), but non-steroidal anti-inflammatory drugs or neuropathic pain medications were prohibited. We didn’t provide any other types of therapy or suggestion including physiotherapy, occupational therapy or night splint.

Participants were allowed to receive a second injection by the same physiatrist within a 6-months follow-up period, which was considered as recurrence.

### Ultrasound-Guided PDI

All ultrasound-guided PDIs were performed by a physiatrist with experience of more than 8,000 cases of ultrasound-guided intervention, using Toshiba Aplio 500 platinum ultrasound system. All participants were positioned with the palm facing upward, and median nerve was identified at the level of proximal inlet (between the pisiform and scaphoid bones). The ultrasound-guided injection was performed using the radial in-plane approach with a 25-gauge needle. After placing the needle between the median nerve and flexor retinaculum, half of the 5% dextrose water (D5W) was injected to separate the two. The other half of the D5W was then applied below and around the median nerve to ensure that it was surrounded by D5W and separated from other tendons within the carpal tunnel. The total D5W injected was either 1, 2 or 4 ml. The syringe was covered with white paper and the participants were asked to close their eyes so they remained unaware of the injection amount. All participants were observed for 10 min after injection for possible immediate complications such as bleeding or worsening paresthesia (Wu et al., 2017a).

### Outcome Assessment

Baseline assessment includes demographics, medical history, symptom duration, lesion side and result of Phalen and Tinel tests. With the outcome assessor blinded to allocation, all participants were assessed with visual analog scale (VAS), Boston Carpal Tunnel Questionnaire (BCTQ) symptom severity scale and functional status scale; the 11-item QuickDASH (disabilities of the arm, shoulder and hand questionnaire), cross-sectional area (CSA) of the median nerve by ultrasound and electrophysiological evaluation at post-injection at the 1st, 4th, 12th, and 24th week.

#### Primary Outcome: VAS

The continuous VAS scale was used to evaluate pain or paresthesia severity as primary outcome. A score of 10 means unbearable, and a score of 0 means no pain/paresthesia at all. We adopted the maximal pain VAS as score measurement.

#### Secondary Outcome: BCTQ and QuickDASH

BCTQ Score is the most commonly used questionnaire for CTS. It includes two subsets of scale, named “symptom severity scale” and “functional status scale (Levine et al., 1993).” The symptom severity scale includes 11 questions, which measure the severity, duration and frequency of daytime and nighttime symptoms, while the functional status scale includes eight questions, which assess the difficulty in performing activities of daily living (ADL). Each question was answered on a scale of 1–5, with 1 being no symptoms or functional disability, and 5 being most severe and unable to perform certain activities. While the original article uses the mean of all the questions, we used the sum of all the questions. QuickDASH measures the performance of ADL, upper extremity discomfort severity, and the severity of hindered sleep and social life (Beaton et al., 2005).

#### Secondary Outcome: CSA of Median Nerve

Ultrasound measurements of the median nerve CSA may be helpful for diagnosis and treatment effect follow-up in CTS (Tai et al., 2012; Wang et al., 2018). At the carpal tunnel inlet level (between the pisiform and scaphoid bones), the CSA was calculated by ultrasound machine after using a caliper to encompass the median nerve manually.

#### Secondary Outcome: Electrophysiological Evaluation

The distal motor latency of the thenar muscles was measured with median nerve stimulation at the wrist at a 7 cm distance; and the orthodromic SNCV was also measured. The detailed method of measurement is the same as described above.

### Statistical Analyses

All data analyses were done using IBM SPSS Statistics Version 22. Because a preliminary Shapiro-Wilks test demonstrated that samples followed a normal distribution, we decided to analyze demographic data between groups with one-way ANOVA for continuous data and Chi-square test for categorical data. Repeated measures ANOVA were used for analyzing the follow-up data compared with the baseline. One-way ANOVA was also used for the comparison between groups during follow-up, for VAS score, BCTQ (symptom severity and functional status), QuickDASH, CSA of median nerve and electrophysiological studies. All statistical tests were two-tailed, and a *p* value of less than 0.05 was considered statistically significant.

## Results

### Clinical Characteristics

A total of 63 wrists (21 wrists in each group) were analyzed. All participants received a completed follow-up of up to 24 weeks after injection. No significant difference was observed between the three groups in all variables, including age, gender, hypertension, diabetes, symptom duration, Phalen or Tinel test positive rate, lesion side, VAS, BCTQ, QuickDASH, parameters of electrodiagnosis or CSA (Table 1).

**TABLE 1:** Baseline characteristics between three groups.

	4 ml group (*n* = 21)	2 ml group (*n* = 21)	1 ml group (*n* = 21)	*p*-Value^a^
Age (SD)	58.4 (9.6)	55.2 (10.7)	60.3 (8.6)	0.231
Female (%)	95.2	81.0	81.0	0.311
Hypertension (%)	28.6	28.6	47.6	0.327
DM (%)	19.0	19.0	28.6	0.692
Symptoms duration (SD)	54.4 (72.3)	20.6 (28.2)	49.8 (60.9)	0.134
Phalen or tinel test positive (%)	42.9	52.4	38.1	0.638
Lesion side (left, %)	45.0	40.0	61.9	0.349
Primary outcome: VAS	5.4 (1.5)	5.9 (2.1)	5.4 (1.8)	0.797
Secondary outcome				
BCTQ (SD)	43.6 (10.4)	40.8 (15.5)	38.4 (14.6)	0.902
BCTQ-S (SD)	24.9 (6.6)	24.1 (9.3)	23.7 (10.0)	0.881
BCTQ-F (SD)	18.7 (5.5)	16.7 (5.8)	14.7 (5.2)	0.134
QuickDASH (SD)	24.3 (6.8)	23.1 (9.9)	22.0 (8.3)	0.666
Electrodiagnosis (SD)				
Motor DL, ms	5.6 (1.6)	5.5 (1.5)	5.4 (1.6)	0.925
SNCV Finger-wrist	30.2 (7.2)	31.5 (7.9)	32.7 (6.6)	0.432
SNCV Palm-wrist	25.1 (4.7)	26.9 (5.8)	26.4 (7.0)	0.572
CSA (SD), mm^2^	14.11 (2.60)	14.8 (3.8)	15.3 (4.4)	0.355

D5W, 5% dextrose water; SD, standard deviation; DM, diabetes mellitus; VAS, visual analog scale; BCTQ, Boston Carpal Tunnel Syndrome Questionnaire (F, function; S, symptom severity); DL, distal latency of median nerve; SNCV, sensory nerve conduction velocity (finger-wrist, finger to wrist segment; palm-wrist, palm to wrist segment); CSA, cross-sectional area of median nerve.

a Between-group comparison: one-way ANOVA for continuous data and chi-square test for categorical data.

### Within-Group PDI Effects

In every group, we observed VAS, BCTQ and QuickDASH significantly improved from baseline data at all follow-up time-points (Table 2 and Supplementary Table S1). For parameters of electrodiagnosis, however, there was no significant difference compared to baseline except for SNCV (finger-wrist) change in the 4-ml group. The median nerve CSA decreased from baseline significantly in both the 2 and 4-ml groups (Supplementary Tables S2,S3).

**TABLE 2:** Primary outcome in the three groups: mean VAS.

VAS	Baseline	1W	4W	12W	24W	*p*-Value^a^
1 ml group	5.38 (1.83)	3.79 (2.37)	4.07 (1.89)	3.69 (1.87)	3.48 (2.36)	<0.001**
2 ml group	5.93 (2.07)	4.51 (2.39)	4.00 (2.74)	3.50 (2.57)	2.79 (2.22)	<0.001**
4 ml group	5.40 (1.51)	3.02 (1.82)	1.88 (1.76)	1.55 (1.91)	2.24 (2.34)	<0.001**

SD, standard deviation; DM, diabetes mellitus; VAS, visual analog scale.

*p < 0.05, **p < 0.01.

a One-way repeated-measures ANOVA was used for within-group analysis. The data was presented as mean (SD).

### Between-Group PDI Effects: Primary Outcome

**FIGURE 2:**
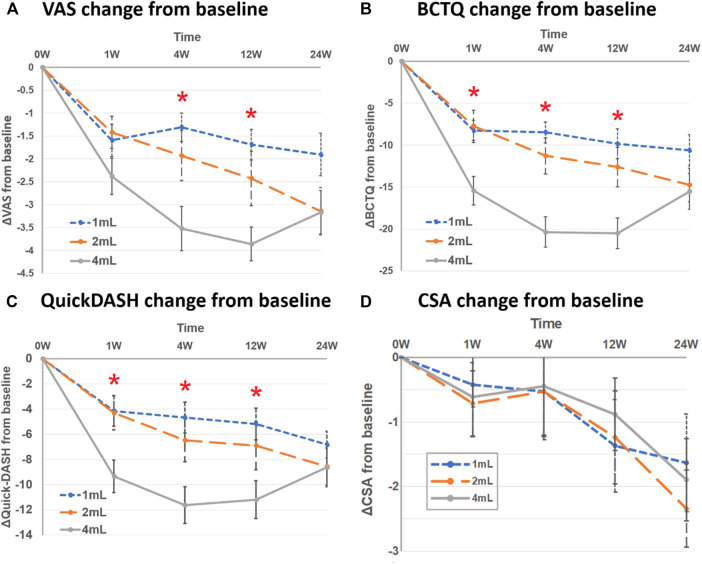
Mean change from baseline between three groups at follow-up time points (1st, 4th, 12th and 24th week) in **(A)** visual analog scale (VAS), **(B)** Boston Carpal Tunnel Syndrome Questionnaire (BCTQ) scores, **(C)** QuickDASH, and **(D)** cross-sectional area of median nerve (CSA). Asterisk represented significant difference between three groups.

In the 4-ml group, the mean change of VAS from baseline showed a greater improvement than the other groups at the 4th week [4 ml: −3.5 (SD 4.9), 2 ml: −1.9 (SD 6.3), 1 ml: −1.3 (SD 2.0)] and 12th week [4 ml: −3.9 (SD 2.9), 2 ml: −2.4 (SD 7.4), 1 ml: −1.7 (SD 2.4)] of post-injection follow-up. There was no significant difference between the three groups at the 1st and 24th weeks of post-injection follow-up (Table 3 and Figure 2).

**TABLE 3:** Comparison of mean change from baseline in outcomes between three groups.

	4 ml group (*n* = 21)	2 ml group (*n* = 21)	1 ml group (*n* = 21)	*p*-Value^a^
Primary outcome ΔVAS				
1 week	−2.38 (1.81)	−1.42 (1.61)	−1.60 (1.63)	0.151
4 weeks	−3.52 (2.22)	−1.93 (2.52)	−1.31 (1.41)	0.003**
12 weeks	−3.86 (1.69)	−2.43 (2.72)	−1.69 (1.54)	0.004**
24 weeks	−3.17 (2.18)	−3.14 (2.37)	−1.90 (2.12)	0.119
Secondary outcome				
ΔBCTQ (SD)				
1 week	−15.43 (8.83)	−7.76 (11.20)	−8.24 (7.09)	0.014*
4 weeks	−20.38 (9.55)	−11.29 (12.95)	−8.48 (6.67)	0.001**
12 weeks	−20.52 (9.81)	−12.62 (14.19)	−9.86 (9.72)	0.010*
24 weeks	−15.52 (12.07)	−14.76 (11.93)	−10.62 (10.55)	0.340
ΔBCTQ-S (SD)				
1 week	−8.57 (4.88)	−4.95 (7.47)	−5.67 (4.88)	0.163
4 weeks	−12.00 (6.90)	−7.52 (8.05)	−5.91 (5.14)	0.015*
12 weeks	−12.10 (6.16)	−8.62 (8.94)	−7.76 (7.48)	0.157
24 weeks	−9.67 (8.22)	−9.91 (7.23)	−7.95 (7.76)	0.674
ΔBCTQ-F (SD)				
1 week	−6.86 (4.14)	−2.81 (4.84)	−2.57 (2.46)	0.001**
4 weeks	−8.38 (4.54)	−3.76 (5.64)	−2.57 (2.48)	0.0002**
12 weeks	−8.43 (5.65)	−4.00 (6.24)	−2.10 (3.32)	0.001**
24 weeks	−5.86 (5.10)	−4.86 (5.69)	−2.67 (3.76)	0.108
ΔQuickDASH (SD)				
1 week	−9.33 (5.89)	−4.29 (6.27)	−4.14 (5.49)	0.008**
4 weeks	−11.62 (6.70)	−6.48 (7.79)	−4.67 (5.67)	0.004**
12 weeks	−11.19 (6.87)	−6.91 (8.73)	−5.19 (5.75)	0.027*
24 weeks	−8.62 (6.90)	−8.57 (7.08)	−6.81 (5.64)	0.599
Electrodiagnosis (SD)				
ΔMotor DL, ms				
1 week	0.06 (0.54)	0.01 (0.88)	−0.33 (0.96)	0.294
4 weeks	0.37 (1.09)	−0.13 (0.58)	−0.08 (0.63)	0.124
12 weeks	0.15 (0.87)	−0.16 (0.62)	−0.08 (0.60)	0.375
24 weeks	0.09 (0.95)	−0.32 (0.72)	−0.09 (0.57)	0.263
ΔSNCV finger-wrist				
1 week	0.45 (3.66)	0.80 (4.63)	0.00 (4.14)	0.841
4 weeks	0.55 (3.03)	1.56 (3.43)	−0.48 (5.16)	0.307
12 weeks	3.11 (3.79)	1.59 (3.15)	0.44 (4.89)	0.138
24 weeks	3.52 (3.71)	2.45 (3.79)	0.40 (4.32)	0.055
ΔSNCV palm-wrist				
1 week	0.69 (3.20)	0.03 (3.53)	0.27 (2.95)	0.816
4 weeks	0.00 (3.54)	0.02 (3.88)	0.26 (3.89)	0.974
12 weeks	1.14 (2.80)	−0.54 (2.72)	0.25 (4.77)	0.370
24 weeks	1.00 (4.11)	0.499 (3.18)	0.12 (5.28)	0.767
ΔCSA (SD), mm^2^				
1 week	−0.61 (2.68)	−0.71 (2.19)	−0.42 (1.50)	0.917
4 weeks	−0.44 (3.31)	−0.53 (3.04)	−0.53 (3.27)	0.996
12 weeks	−0.88 (2.45)	−1.24 (3.15)	−1.37 (3.13)	0.879
24 weeks	−1.89 (2.28)	−2.34 (2.96)	−1.63 (2.87)	0.720

D5W, 5% dextrose water; SD, standard deviation; DM, diabetes mellitus; VAS, visual analog scale; BCTQ, Boston Carpal Tunnel Syndrome Questionnaire (F, function; S, symptom severity); DL, distal latency of median nerve; SNCV, sensory nerve conduction velocity (finger-wrist, finger to wrist segment; palm-wrist, palm to wrist segment); CSA, cross-sectional area of median nerve.

*p < 0.05, **p < 0.01.

a Between-group comparison: one-way ANOVA was used for statistical analysis.

### Between-Group PDI Effects: Secondary Outcome

In the 4-ml group, the mean change of BCTQ from baseline improved more compared to other groups at the 1st week [4 ml: −15.4 (SD 8.8) vs. 2 ml: −7.8 (SD 11.2) vs. 1 ml: −8.2 (SD 7.1)], 4th week [4 ml: −20.4 (SD 9.5) vs. 2 ml: −11.3 (SD 12.9) vs. 1 ml: −8.5 (SD 6.7)] and 12th week [4 ml: −20.5 (SD 9.8) vs. 2 ml: −12.6 (SD 14.2) vs. 1 ml: −9.9 (SD 9.7)] post-injection. There was no significant difference, however, between the three groups at the 24th week post-injection (Table 3 and Figure 2).

For the mean change of QuickDASH from baseline, similar to BCTQ results, the 4-ml group yielded more improvement than other groups at the 1st, 4th and 12th weeks post-injection, but not at the 24th week (Table 3 and Figure 2).

As for the parameters of electrodiagnosis (distal motor latency and SNCV) and median nerve CSA, there was no significant difference between the three groups at any follow-up time points (Table 3 and Figure 2).

### Recurrence Rate

The proportion of participants who received a second injection within a 6-months follow-up period was 23.8% in 1-ml group, 23.8% in 2-ml group, and 28.6% in 4-ml group. No significant difference was observed among the three groups (*p* = 0.952).

### Adverse Effects

No severe adverse effects, such as infection, persistent bleeding, hematoma or death, were identified in any groups during the follow-up period. No significant difference in the minor symptoms and neuropathic symptoms was observed among the three groups (all *p* > 0.05) (Table 4).

**TABLE 4:** Adverse effect.

	4 ml group	2 ml group	1 ml group	*p*-Value^a^
Transient, %				
Minor symptoms^b^	45.0	20.0	23.8	0.180
Neuropathic symptoms^c^	20.0	20.0	9.5	0.589
Severe complication^d^, %	0	0	0	1.000

D5W, 5% dextrose water.

a Between-group comparison: chi-square test for categorical data analysis.

b Minor symptoms included needling pain, subjective swelling or dizziness subsided within 10 min.

c Neuropathic symptoms included electric shock sensation or finger numbness subsided within 10 min.

d Severe complication included infection, persisted bleeding, hematoma or death.

## Discussion

In this RCT, ultrasound-guided PDI with 1, 2 or 4 ml D5W could all significantly improve pain and function for CTS. The 4-ml group yielded better VAS reduction at the 4th and 12th week and functional enhancement at the 1st, 4th, and 12th week post-injection, compared to the 1 and 2-ml groups. No significant between-group differences were observed in parameters of electrophysiological study or median nerve CSA at any follow-up time points. There were no severe complications, and transient minor adverse effects occurred similarly in all three groups.

Our study focused on the efficacy of PDI and showed that 4 ml dextrose injection is superior to that of 1 or 2 ml with regard to pain and change in functional outcome. Nevertheless, all three groups benefited from reduced pain and improved function within the 6-months follow-up period despite injection volume. Previous studies showed promising effects of dextrose within 6 months (Wu et al., 2017b) and even better than steroids at 4–6 months post-injection (Wu et al., 2018). The placebo group, however, showed reduced symptoms with saline injection as well; therefore, volume effect should be addressed. To investigate the efficacy of hydrodissection, Bland et al. demonstrated the complicated and multifactorial pathogenesis of CTS comprised external compression to the nerve and internal ischemia/inflammation-induced fibrosis to surrounding soft tissues (Bland and Rudolfer, 2003). Increased subsynovial connective tissue in the carpal tunnel affected nerve compliance and permeability (Ettema et al., 2004), and correlated with clinical symptom severity (Tat et al., 2015). A systematic review documented reduced nerve excursion in CTS people compared to healthy controls (Ellis et al., 2017). One cadaver study showed decreased gliding resistance of the nerve in the carpal tunnel after hydrodissection (Evers et al., 2018). Two clinical RCTs further showed positive effects of hydrodissection on pain, function and CSA (Roghani et al., 2018; Wu et al., 2019). Our study adopted an identical injection drug (5% dextrose) to eliminate confounding bias of dextrose. We validated that even 1 ml PDI would be effective for CTS, despite 4 ml yielding more improvement. The results were compatible with our experience during injection where we observed further expansion of the perineural space and longitudinal drug spreading. Therefore, we believe that volume effect did matter for PDI.

In this study, three groups presented improved pain and function comparing to baseline; nevertheless, only the 1 and 2-ml groups exhibited trends of decreased VAS, BCTQ and QuickDASH up to the 6th month of the follow-up period instead of the 4-ml group (Figure 2). Although the effect of dextrose, conducted by a previous RCT, lasted for up to 6 months (Wu et al., 2017b), the effect of perineural saline injection showed an improved BCTQ only until the 3rd month post-injection which deteriorated by the 6th month of follow-up (Wu et al., 2019). Similarly, another RCT reported the improvement of BCTQ did not last until the 6th month after hydrodissection (with either steroid or hyaluronidase) (Alsaeid, 2019). In this study, via post-hoc Bonferroni analysis, pain and functional outcomes were not significant between the 3rd and 6th months follow-up in the 1, 2 and 4-ml groups (VAS: *p* = 1.000, 0.895, 0.750, respectively; BCTQ: *p* = 1.000, 1.000, 0.295, respectively). Further researches with longer follow-up are necessary to investigate whether the effect of dextrose hydrodissection persists.

We observed significantly reduced CSA of the median nerve by the 6-months follow-up in the 2 and 4-ml groups, instead of improvement in electrophysiological parameters. Many studies investigated different injectates (including steroid, dextrose and platelet-rich plasma) to decrease CSA after injection, even with placebo saline (Wu et al., 2017b; Wang et al., 2018; Shen et al., 2019; Wu et al., 2019). Although not significant, the 1-ml group of our study showed the CSA exhibited a trend of reduction of intraneural inflammation, edema, or swelling of subsynovial connective tissue, which aligns with a previous hypothesis posed by Wang et al. (2018). With the randomization and use of identical injectate, we could infer the volume of hydrodissection mattered.

Although the participants of the 4-ml group complained of minor symptoms, there were no severe adverse effects in any groups. Old studies documented median nerve injury and tendon ruptures after multiple injections (Gottlieb and Riskin, 1980; Kasten and Louis, 1996); however, contemporary ultrasound-guided techniques escalated the accuracy of injection, and were shown to be more effective than blind injection (Lee et al., 2014; Chen et al., 2015). No severe complications were noted in the studies who used the aforementioned ultrasound-guided median nerve injection. While the 4-ml injection delivered more volume of dextrose into the carpal tunnel, potentially resulting in transient higher pressure that may explain the minor symptoms, no participants in the 4-ml group asked for termination of injection.

Although recurrence rate in 4-ml group was slightly higher than other two groups, no significant difference was observed among the three groups within a 6-months follow-up period. Recent research revealed multiple PDI with high volume dextrose up to 10 ml exerted longer cumulative effects, comparing to their previous study with 5 ml single PDI (Li et al., 2020). In our study, we hypothesize the longer symptom duration and motor distal latency of electrodiagnosis in 4-ml group might cause slightly higher recurrence rate than other groups [symptom duration (months): 54.4 in 4-ml group, 20.6 in 2-ml group and 49.8 in 1-ml group; motor distal latency (ms): 5.6 in 4-ml, 5.5 in 2-ml and 5.4 in 1-ml]. Aforementioned study exhibited similar PDI results that severe CTS patients had less excellent outcome than mild to moderate CTS patients (Li et al., 2020).

There were several limitations to this study. First, we did not exclude severe CTS patients (Chang et al., 2008). Though we believed randomization would minimize potential bias (19.0, 14.3, 14.3% in 1, 2 and 4-ml groups, respectively; *p* = 0.918), the severity of CTS may have obscured the effect of injection. Second, we enrolled participants with bilateral hands, where one hand might influence the functional outcomes of the other hand. Third, this study did not provide direct evidence that larger volume reduced more adhesion around the median nerve. Further studies on the median nerve excursion after PDI are warranted.

## Conclusion

Ultrasound-guided PDI with 4 ml D5W provided better efficacy in symptom relief and functional improvement for CTS than with 1 and 2 ml at the 1st, 4th, and 12th week post-injection, with no reports of severe adverse effects. There was no significant difference between the three groups at the 24th-week post-injection follow-up.

## Data Availability Statement

The raw data supporting the conclusions of this article will be made available by the authors, without undue reservation.

## Ethics Statement

The studies involving human participants were reviewed and approved by National Taiwan University Hospital, Taiwan. The patients/participants provided their written informed consent to participate in this study.

## Author Contributions

M-TL, C-LL, and C-HW contributed to research design and conception. M-TL and C-LL analyzed data. M-YH, H-WH, and C-CC helped interpreting the results. M-TL drafted the first edition of the manuscript. All authors revised, and agreed the manuscript.

## Funding

The research was funded by National Taiwan University Hospital (NTUH 108-M4366, 109-M4670) and Ministry of Science and Technology, Taiwan (MOST 107-2314-B-002-045-MY3, MOST 107-2314-B-002-232-MY3).

## Conflict of Interest

The authors declare that the research was conducted in the absence of any commercial or financial relationships that could be construed as a potential conflict of interest.
