# Supplementary material for: Volume Matters in Ultrasound-Guided Perineural Dextrose Injection for Carpal Tunnel Syndrome: A Randomized, Double-Blinded, Three-Arm Trial
Source: Front Pharmacol. 2020 Dec 17;11:625830. doi: 10.3389/fphar.2020.625830 (PMC7773892; doi:10.3389/fphar.2020.625830)
Supplement: Supplementary file 1 [file datasheet1.docx]

| **Supplementary Table 1.** Secondary outcome in three groups: mean BCTQ and QuickDASH | | | | | | |
| --- | --- | --- | --- | --- | --- | --- |
|  | **baseline** | **1W** | **4W** | **12W** | **24W** | ***p*-value^†^** |
| **BCTQ** |  |  |  |  |  |  |
| 1mL group | 38.38 (14.60) | 30.14 (13.40) | 29.90 (13.64) | 28.52 (9.81) | 27.76 (9.25) | **<0.001**** |
| 2mL group | 40.76 (15.48) | 33.00 (14.21) | 29.48 (13.33) | 28.14 (12.01) | 26.00 (10.44) | **<0.001**** |
| 4mL group | 43.57 (10.41) | 28.14 (8.49) | 23.19 (4.24) | 23.05 (4.79) | 28.05 (11.15) | **<0.001**** |
| **QuickDASH** |  |  |  |  |  |  |
| 1mL group | 22.00 (8.28) | 17.86 (8.33) | 17.33 (8.33) | 16.81 (6.19) | 15.19 (5.28) | **<0.001**** |
| 2mL group | 23.14 (9.91) | 18.86 (8.62) | 16.67 (7.69) | 16.24 (7.82) | 14.57 (6.01) | **<0.001**** |
| 4mL group | 24.33 (6.76) | 15.00 (4.29) | 12.71 (3.04) | 13.14 (3.37) | 15.71 (6.73) | **<0.001**** |
| † One-way repeated-measures ANOVA was used for within-group analysis.  The data was presented as mean (SD).  Abbreviation: SD, standard deviation; BCTQ, Boston Carpal Tunnel Syndrome Questionnaire.  *p < 0.05, **p < 0.01. | | | | | | |

| **Supplementary Table 2.** Secondary outcome in three groups: electrodiagnosis parameters | | | | | | |
| --- | --- | --- | --- | --- | --- | --- |
| **Electrodiagnosis** | **baseline** | **1W** | **4W** | **12W** | **24W** | ***p*-value^†^** |
| **Motor DL** |  |  |  |  |  |  |
| 1mL group | 5.42 (1.64) | 5.08 (1.73) | 5.34 (1.77) | 5.34 (1.81) | 5.33 (1.80) | 0.368 |
| 2mL group | 5.48 (1.53) | 5.51 (1.98) | 5.36 (1.34) | 5.32 (1.37) | 5.17 (1.53) | 0.413 |
| 4mL group | 5.58 (1.62) | 5.69 (1.71) | 5.94 (2.15) | 5.73 (1.90) | 5.67 (1.97) | 0.421 |
| **SNCV finger-wrist** |  |  |  |  |  |  |
| 1mL group | 32.67 (6.59) | 32.67 (8.11) | 32.19 (7.43) | 33.11 (6.16) | 33.07 (6.29) | 0.880 |
| 2mL group | 31.51 (7.86) | 32.31 (7.57) | 33.00 (6.09) | 33.10 (6.41) | 33.96 (6.05) | 0.064 |
| 4mL group | 30.19 (7.20) | 30.64 (6.35) | 30.88 (6.08) | 33.09 (6.17) | 33.71 (5.52) | **<0.001**** |
| **SNCV palm-wrist** |  |  |  |  |  |  |
| 1mL group | 26.39 (7.00) | 26.66 (7.09) | 26.64 (5.01) | 26.64 (5.71) | 26.51 (5.88) | 0.997 |
| 2mL group | 26.94 (5.76) | 26.97 (5.55) | 26.77 (4.77) | 26.40 (5.44) | 27.93 (5.30) | 0.352 |
| 4mL group | 25.07 (4.69) | 25.76 (4.11) | 24.92 (4.92) | 26.13 (4.36) | 26.07 (5.90) | 0.365 |
| † One-way repeated-measures ANOVA was used for within-group analysis.  The data was presented as mean (SD).  Abbreviation: SD, standard deviation; BCTQ, Boston Carpal Tunnel Syndrome Questionnaire; DL, distal latency of median nerve; SNCV, sensory nerve conduction velocity (finger-wrist, finger to wrist segment; palm-wrist, palm to wrist segment).  *p < 0.05, **p < 0.01. | | | | | | |

| **Supplementary Table 3.** Secondary outcome in three groups: mean CSA | | | | | | |
| --- | --- | --- | --- | --- | --- | --- |
| **CSA** | **baseline** | **1W** | **4W** | **12W** | **24W** | ***p*-value^†^** |
| 1mL group | 15.26 (4.40) | 14.84 (4.56) | 14.74 (4.54) | 13.89 (4.31) | 13.63 (4.25) | 0.111 |
| 2mL group | 14.82 (3.78) | 14.11 (3.70) | 14.42 (3.78) | 13.58 (3.66) | 12.47 (3.39) | **0.001**** |
| 4mL group | 14.11 (2.60) | 13.21 (2.44) | 13.05 (2.86) | 13.26 (2.54) | 12.21 (2.23) | **0.018*** |
| † One-way repeated-measures ANOVA was used for within-group analysis.  The data was presented as mean (SD).  Abbreviation: SD, standard deviation; BCTQ, Boston Carpal Tunnel Syndrome Questionnaire; CSA, cross-sectional area of median nerve.  *p < 0.05, **p < 0.01. | | | | | | |
